# Magnetic resonance-guided focused ultrasound unilateral thalamotomy for medically refractory essential tremor: 3-year follow-up data

**DOI:** 10.3389/fneur.2024.1360035

**Published:** 2024-04-26

**Authors:** Stefano Tamburin, Fabio Paio, Tommaso Bovi, Giorgia Bulgarelli, Michele Longhi, Roberto Foroni, Elisa Mantovani, Paolo Maria Polloniato, Micaela Tagliamonte, Emanuele Zivelonghi, Chiara Zucchella, Carlo Cavedon, Antonio Nicolato, Benedetto Petralia, Francesco Sala, Bruno Bonetti, Michele Tinazzi, Stefania Montemezzi, Giuseppe Kenneth Ricciardi

**Affiliations:** ^1^Neurology Unit, Department of Neurosciences, Azienda Ospedaliera Universitaria Integrata, Verona, Italy; ^2^Neurology Section, Department of Neurosciences, Biomedicine, and Movement Sciences, University of Verona, Verona, Italy; ^3^Stereotactic Neurosurgery and Radiosurgery Unit, Department of Neurosciences, Azienda Ospedaliera Universitaria Integrata, Verona, Italy; ^4^Medical Physics Unit, Department of Pathology and Diagnostics, Azienda Ospedaliera Universitaria Integrata, Verona, Italy; ^5^Neuroradiology Unit, Department of Pathology and Diagnostics, Azienda Ospedaliera Universitaria Integrata, Verona, Italy; ^6^Neurosurgery Unit, Department of Neurosciences, Azienda Ospedaliera Universitaria Integrata, Verona, Italy; ^7^Neurosurgery Section, Department of Neurosciences, Biomedicine, and Movement Science, University of Verona, Verona, Italy; ^8^Radiology Unit, Department of Pathology and Diagnostics, Azienda Ospedaliera Universitaria Integrata, Verona, Italy

**Keywords:** functional neurosurgery, MRgFUS, non-invasive, thalamus, thalamotomy, tremor

## Abstract

**Introduction:**

Magnetic resonance–guided focused ultrasound (MRgFUS) thalamotomy of the ventralis intermediate (Vim) nucleus is an “incisionless” treatment for medically refractory essential tremor (ET). We present data on 49 consecutive cases of MRgFUS Vim thalamotomy followed-up for 3 years and review the literature on studies with longer follow-up data.

**Methods:**

A retrospective chart review of patients who underwent MRgFUS thalamotomy (January 2018–December 2020) at our institution was performed. Clinical Rating Scale for Tremor (CRST) and Quality of Life in Essential Tremor (QUEST) scores were obtained pre-operatively and at each follow-up with an assessment of side effects. Patients had post-operative magnetic resonance imaging within 24 h and at 1 month to figure out lesion location, size, and extent. The results of studies with follow-up ≥3 years were summarized through a literature review.

**Results:**

The CRST total (baseline: 58.6 ± 17.1, 3-year: 40.8 ± 18.0) and subscale scores (A + B, baseline: 23.5 ± 6.3, 3-year: 12.8 ± 7.9; C, baseline: 12.7 ± 4.3, 3-year: 5.8 ± 3.9) and the QUEST score (baseline: 38.0 ± 14.8, 3-year: 18.7 ± 13.3) showed significant improvement that was stable during the 3-year follow-up. Three patients reported tremor recurrence and two were satisfactorily retreated. Side effects were reported by 44% of patients (severe: 4%, mild and transient: 40%). The improvement in tremor and quality of life in our cohort was consistent with the literature.

**Conclusion:**

We confirmed the effectiveness and safety of MRgFUS Vim thalamotomy in medically refractory ET up to 3 years.

## Introduction

First ablative magnetic resonance–guided focused ultrasound (MRgFUS) thalamotomy dates to around 15 years ago, although its use has experienced exponential growth in recent years (1). MRgFUS is an “incisionless” technique that uses ultrasound from an array of transducers around the skull to induce focal thermal ablation lesions in the brain during an awake outpatient procedure and magnetic resonance imaging (MRI) for target definition, treatment planning, and closed-loop control of energy deposition (2, 3).

MRgFUS is applied to patients with medically refractory essential tremor (ET), who are not suitable for or refuse an invasive surgical procedure, to target the ventralis intermediate (Vim) nucleus of the thalamus (2). A randomized controlled trial showed that unilateral MRgFUS Vim thalamotomy may induce nearly 50% reduction in contralateral tremor in patients with moderate to severe medically refractory ET 1 year after treatment (4) and a sustained clinical benefit at 2 years (2). The benefit up to 1 year has been confirmed by many studies and summarized in two systematic reviews (5, 6) and some reports confirmed the positive effect at 2-year follow-up according to a meta-analysis with meta-regression (6). Only few studies explored the MRgFUS thalamotomy outcomes at longer time points, i.e., 3- (7, 8), 4- (9), and 5-year follow-ups (10, 11) in ET, offering a less definitive scenario of its longer-term benefit.

Factors that influence MRgFUS outcome include skull density ratio [SDR; (2)], lesion location and volume (12–14), patient age, disease duration, peak temperature, and number of sonications (15).

The aim of this study is two-fold. The first aim is to report data of a retrospective single-center observational study from our Institution’s experience with MRgFUS Vim thalamotomy in patients with medically refractory unilateral ET followed-up over a period of 3 years. Our data may offer a “real-world” clinical experience to confirm the clinical efficacy of this procedure and help to identify areas for future research. The second aim is to summarize the results of studies with follow-up of at least 3 years through a discussion of the literature.

## Methods

### Subjects

We retrospectively reviewed prospectively collected data of 49 patients, who were consecutively treated between January 2018 and December 2020 at Verona University Hospital, Verona, Italy. Therefore, inclusion and exclusion criteria align with the eligibility criteria for MRgFUS thalamotomy. We treated adult patients (>18 y/o) with disabling ET unresponsive to at least two classes of medication, who could tolerate and cooperate during the procedure and were unwilling or ineligible for deep brain stimulation. Exclusion criteria included general MRI contraindications, impossibility to avoid sonication of sensitive brain/skull structures, SDR value <0.40, patients on anticoagulant and/or antiplatelet therapy with no possibility of temporary suspension, and those with significant and active comorbidities.

All patients signed an informed consent before MRgFUS thalamotomy and provided a specific informed consent to participate in the observational study, delivered either upon admission to the Hospital or during one of the follow-up visits. The study was conducted according to the Declaration of Helsinki and approved by the local ethical committee (Ethical Committee of the Veneto Region South-West Area at the Verona University Hospital – CET-ASOV; approval number 133CET).

A detailed chart review was performed to extract demographics (age, gender), disease characteristics (ET duration, baseline ET severity and quality of life, treated side), and radiological parameters such as SDR and lesion volume at the 1-month MRI. As previous described by other authors (16), volume was determined based on the 2-mm slice axial and coronal T2-weighted images, considering the three maximum diameters [latero-lateral (x), anterior-posterior (y), and cranio-caudal (z)] and estimated by using the ellipsoid approximation formula: 4/3 × π × (x/2) × (y/2) × (z/2).

### MRgFUS procedure

All patients underwent a prophylaxis protocol with corticosteroids, with the administration of intravenous dexamethasone 4 mg every 8 h on the day of the thalamotomy (i.e., one administration before and two administrations after the procedure) followed by slow tapering with oral prednisone in the next 2–3 weeks.

Details of the MRgFUS procedure have been previously published elsewhere (2, 4). Briefly, the patient’s head was shaved, and a modified stereotactic frame was affixed on the patient’s skull after infiltration with local anesthetic. A flexible rubber gasket was placed over the frame and the patient’s head rigidly fixed to the MRI table. The space between the patient’s head and the MRgFUS transducer was filled with circulating, degassed water and T2-weighted MRI images were obtained in sagittal, coronal, and axial planes. Standard stereotactic coordinates were used to locate the thalamic Vim nucleus, i.e., X: 11 mm from the lateral wall of third ventricle, Y: average of one third/one fourth distance of the anterior commissure-posterior commissure (AC-PC) distance in front of the PC, Z: 1–2 mm above the intercommissural plane. Minor corrections to the initial target were made to adjust for individual patient anatomy. The sonication procedure, i.e., the administration of thermal energy to the brain target by the array of ultrasound transducers, consists of several phases (1, 17). In the alignment phase, brief low-energy sonications aim to reach a temperature of approximately 40–45°C without biological effects and thermometric maps are acquired to confirm the accuracy of the sonication point. The verification phase involves sonications reaching higher temperatures (46–54°C) for neuromodulation and testing potential adverse events. In the verification phase, serial neurological examinations allow for probing the magnitude of postural and intentional tremor response (writing, spiral and line drawing, drinking from a bottle) and to assess possible side effects (motor and sensory function, speech, coordination). Once the ‘sweet spot’ that maximizes clinical benefit and cuts adverse effects has been found, the procedure moves to the final ablation phase, which involves modulating the energy to achieve effective temperatures for coagulative necrosis (55–60°C) leading to an irreversible lesion. Each phase can be repeated to ensure correspondence between target coordinates and the focal point, check for adverse effects, and confirm effectiveness in treating the patient’s tremor.

### Clinical assessment

Clinical assessments were performed at baseline (T0), and 1 (T1), 3 (T2), 6 (T3), 1 year (T4), 2 years (T5) and 3 years (T6) after the treatment. Tremor was evaluated with the Clinical Rating Scale for Tremor (CRST) (18), which measures the severity of resting, postural and intention tremor (Part A), the severity of upper limb intention tremor during writing, drawing and pouring (Part B), the functional disability related to tremor (Part C) and the subjective % of tremor improvement. Quality of life was explored with the Quality of Life in Essential Tremor (QUEST) questionnaire (19). Side effects and their duration were also recorded. Outcome measures of the treated side were CRST part A and B score, while overall outcome measures included CRST part C and total score, and QUEST score.

### Statistics

Statistical analysis was performed with SPSS version 21.0 (SPSS, Chicago, United States). For continuous variables, normality of distribution was tested with the Shapiro-Wilks test. Differences in outcome measures (CRST, QUEST) at various assessment timepoints were analyzed with repeated measures ANOVA with Greenhouse–Geisser correction (within-group variable: time, T0-T6 for CRST A, B, C and total and QUEST; time, T1-T6 for CRST subjective improvement that was not administered to baseline) followed by post-hoc paired Student’s t-test in case of normal distribution, or the non-parametric Wilcoxon signed-rank order test when the distribution was not normal. *p* < 0.05 (two-tailed, with Bonferroni’s correction as needed) was the significance threshold for all the tests.

### Review of studies with follow-up of at least 3 years

To integrate data derived from the systematic review and meta-analysis by Miller et al. (6) and provide an updated overview (i.e., from 2019 onwards) of the outcomes of unilateral MRgFUS thalamotomy in medically refractory ET, we searched studies with long-term follow-ups (i.e., ≥ 3 years). PubMed/MEDLINE was consulted using the following search string: (“magnetic resonance guided focused ultrasound” OR “MRgFUS” OR “focused ultrasound”) AND (“essential tremor”). Studies were considered eligible if they included measures of ET severity (e.g., CRST) or ET impact on quality of life (e.g., QUEST) assessed prior to and at regularly scheduled follow-up intervals after MRgFUS intervention.

## Results

Baseline demographic and clinical characteristics of the overall patients and those with 3-year follow-up data and treatment parameters are reported in Table 1.

**Table 1:** Demographic and baseline clinical characteristics of the patients and treatment parameters.

Variable	Overall patients (*N* = 49)	Patients with 3-year follow-up (*N* = 35)
Demographic characteristics		
Sex (M/F)	30/19	21/14
Age (years)	72.8 ± 6.9, 73, 49–85	72.7 ± 7.2, 74, 49–85
Baseline clinical characteristics		
ET duration (years)	22.7 ± 14.1, 20, 5–60	22.7 ± 13.0, 20, 5–55
CRST part A, treated side	9.0 ± 3.0, 10, 3–18	8.5 ± 3.7, 9, 4–15
CRST part B, treated side	15.4 ± 4.1, 16, 5–20	15.4 ± 4.2, 17, 5–20
CRST part C	12.7 ± 4.3, 12.5, 4–24	12.2 ± 4.3, 12, 4–24
CRST total severity	58.9 ± 17.1, 59, 22–94	57.5 ± 18.3, 55, 22–94
Quality of life (QUEST)	38.0 ± 14.8, 34, 14–78	37.2 ± 15.7, 32, 14–78
Treated side (R/L)	45/4	31/4
Treatment parameters		
SDR	0.58 ± 0.09, 0.56, 0.41–0.75	0.57 ± 0.10, 0.55, 0.41–0.75
Number of sonications	12.0 ± 3.4, 12, 7–19	11.8 ± 4.2, 11, 7–19
Max temperature (°C)	57.7 ± 1.8, 57, 55–62	57.4 ± 2.0, 57, 55–61
Lesion volume (mm^3^) at 1 month	11.9 ± 17.9, 5.0, 1.3–83.7	12.1 ± 18.5, 6.3, 1.3–83.7

Data are reported as mean ± SD, median, range for continuous variables. CRST, clinical rating scale for tremor; ET, essential tremor; L, left; R, right, QUEST, quality of life in essential tremor; SDR, skull density ratio.

**Figure 1:**
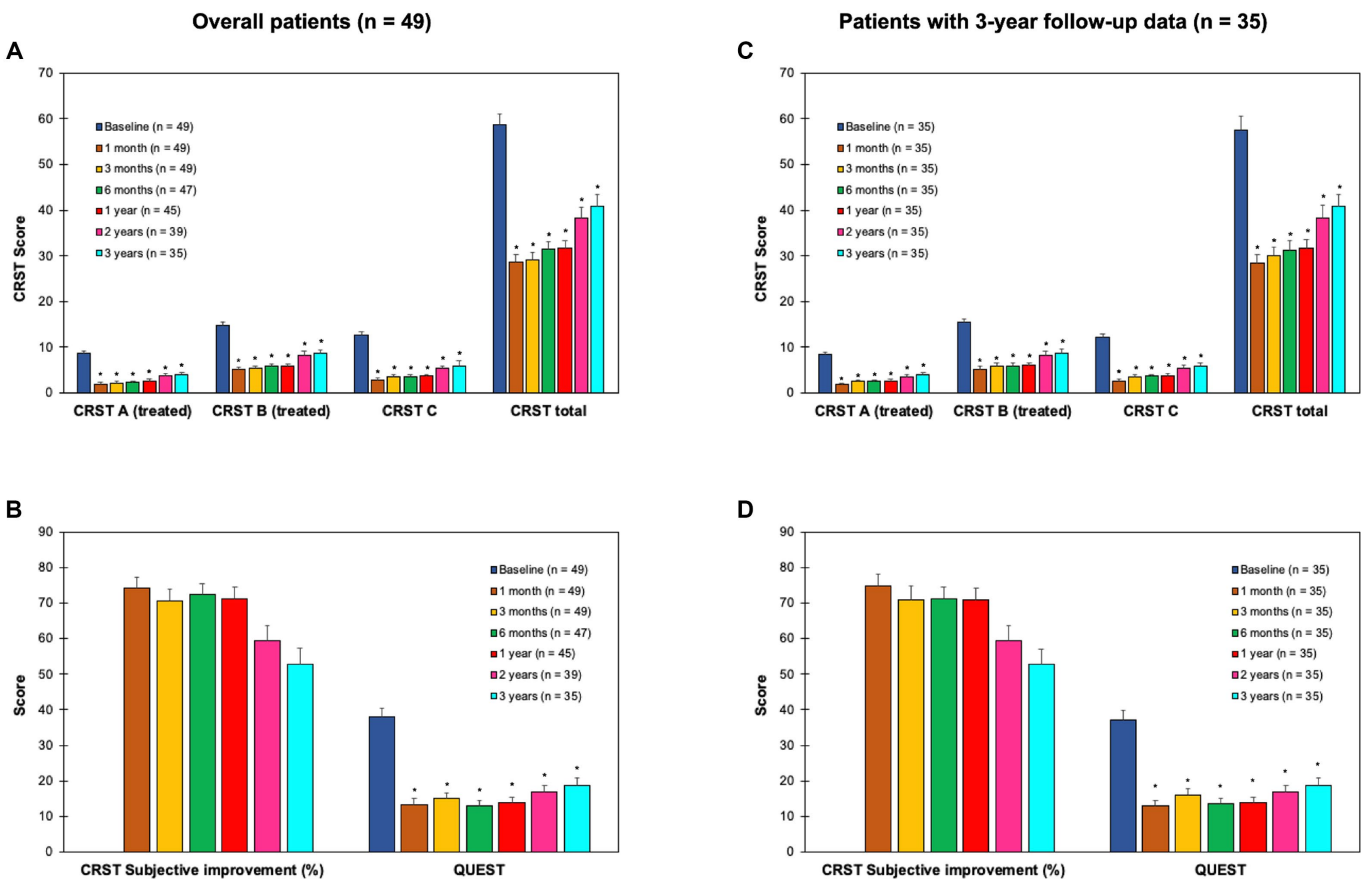
Clinical outcome measures at baseline (T0), and 1 (T1), 3 (T2), 6 months (T3), 1 year (T4), 2 years (T5) and 3 years (T6) after the treatment in objective (upper panels) and subjective measures (lower panels) of tremor in the overall population of patients (*n* = 49; panels **A,B**) and in the patients with 3-year follow-up data (*n* = 35; panels **C,D**). CRST, clinical rating scale for tremor (18) that is composed by part A (tremor severity, treated side), part B (upper limb intentional tremor severity, treated side), part C (functional disability related to tremor; panels **A,C**), and subjective improvement % (panels **B,D**). QUEST: quality of life in essential tremor questionnaire (19) (panels **B,D**). *p < 0.008 (i.e., Bonferroni’s corrected *p*) for T1-T6 vs. T0 comparisons. CRST subjective improvement was stable across time (i.e., *post-hoc* comparisons not significant for T2-T6 vs. T1). Error bars equal 1 SEM.

The CRST (repeated measures ANOVA: CRST A; *F* = 84.1, *p* < 0.001; CRST B: *F* = 30.0, *p* < 0.001; CRST C: *F* = 61.7, *p* < 0.001; CRST total: *F* = 53.4, *p* < 0.001) and the QUEST score (repeated measures ANOVA: *F* = 34.7, *p* < 0.001) showed a significant and consistent improvement of the treated side outcome measures (tremor severity: CRST A: 54–77% across different follow-ups vs. T0, *t* = 9.4–17.7, *p* < 0.001; CRST B, 41–65%, *t* = 5.5–16.2, *p* < 0.001), overall tremor outcome measures (impairment due to tremor: CRST C, 55–78% across different follow-ups vs. T0, *t* = 10.2–14.7, *p* < 0.001; overall tremor score: CRST total, 31–50%, *t* = 7.1–18.4, *p* < 0.001), and quality of life (QUEST: 51–66% across different follow-ups vs. T0, *t* = 6.0–12.2, *p* < 0.001) that was stable in comparison to baseline during the three-year follow-up period (Figure 1). Subjective improvement (CRST subjective: range, 53–74%) showed a reduction over time (repeated measures ANOVA: *F* = 5.3, *p* = 0.01) that was not significant across different follow-ups vs. T1 (*t* = 1.0–3.2, n.s.; Figure 1).

At 1-year follow-up, 3 patients reported loss of benefit with <30% CRST overall score reduction, while 2 and 1 additional patients reported recurrence of tremor with less than 30% benefit on CRST score at 2- and 3-year follow-up, respectively. Two patients were retreated after 13 and 35 months, respectively, with CRST reduction >50%. In one of the retreated patients, the lesion after first treatment was undetectable in T2-weighted images, while the first lesion size was within normal range in the other retreated patient.

Severe and mild side effects are reported in Table 2. Severe side effects included ballism lasting up to 36 months and hemiparesis lasting 1–24 months. The most common mild side effect was ataxia that was short-lasting (i.e., 2 weeks- 3 months) in all cases, except one who reported partial amelioration after 1 month, but persistence up to 1 year. Other mild and transient side effects included short-lasting (i.e., 2 weeks), dysarthria and paresthesia, corticosteroid-related effects (overall, *N* = 3; nocturnal restlessness, *N* = 1; annoying hiccups, *N* = 1; mild transitory hyperglycemia; *N* = 2) that were limited to the corticosteroid administration and then vanishing, and subjective cognitive impairment, without changes to standard neuropsychological testing.

**Table 2:** Side effects to treatment and their duration.

Side effect	Duration
Severe	
Ballism (*N* = 1)	36 months
Hemiparesis (*N* = 3)	1–24 months
Mild	
Ataxia (*N* = 14)	15 days-12 months
Subjective cognitive impairment (*N* = 1)	3 months
Dysarthria (*N* = 4)	15 days
Paresthesia (*N* = 3)	7–15 days
Corticosteroid related (*N* = 3)	15 days-1 month

Five studies were identified that provided data at 3, 4, and 5-year follow-ups (see Table 3 for details).

**Table 3:** MRgFUS thalamotomy studies providing long-term clinical data (i.e., follow-up ≥3 years).

Ref.	Study design	Site (s)	Sample size		FU duration	ET severity (CRST)						QoL (QUEST)	
						CRST part A + B (hand tremor-motor score)		CRST part C		CRST total			
			BL	FU		BL	FU	BL	FU	BL	FU	BL	FU
Halpern et al. (7)^#^	Prospective, controlled, multicenter clinical trial	USA Canada Japan South Korea	*N* = 76 (M: 52, F: 24; age: 71.0 ± 3.8)	*N* = 52	3 y	20.1 ± 4.7	9.5 ± 5.4	16.4 ± 4.6	7.5 ± 6.1	NR	NR	43.1 ± 18.3	23.8 ± 19.6
Peters et al. (8)	Prospective, monocenter clinical trial	Australia	*N* = 30 (M: 23, F: 7; age: 74.5 ± 7.53)	*N* = 6	3 y	21.2 (12.5–30.0)^§^	8.6 (0.2–17.1)^§^	NR	NR	43.8 (21.3–66.4)^§^	23.3 (1.2–45.4)^§^	43.8 (21.3–66.4)^§^	23.3 (1.2–45.4)^§^
Park et al. (9)	Randomized, controlled, monocenter clinical trial	South Korea	*N* = 15	*N* = 12 (M: 10, F: 2; age: 61.7 ± 8.1)	3 y 4 y	17.4 ± 3.8	3 y: 7.5 ± 5.3 4 y: 7.7 ± 4.1	12.7 ± 3.0	3 y: 4.4 ± 3.3 4 y: 4.7 ± 3.0	NR	NR	NA	NA
Cosgrove et al., (10)^#^	Long-term, multicenter, postinterventional clinical trial	USA Canada Japan South Korea	*N* = 76 (M: 52, F: 24; age: 71.0 ± 3.8)	*N* = 52 (3 y) *N* = 45 (4 y) *N* = 40 (5 y; M: 30, F: 10; age: 75 ± 8.4)	3 y 4 y 5 y	20 ± 4.7	3 y: 9.5 ± 5.4 4 y: 9.6 ± 5.8 5 y: 11.0 ± 6.5	16 ± 4.6	3 y: 7.5 ± 6.1 4 y: 8.4 ± 6.9 5 y: 8.9 ± 6.6	NR	NR	43 ± 18	3 y: 26 ± 21 4 y: 28 ± 19 5 y: 30 ± 20
Sinai et al. (11)	Prospective, monocenter clinical trial	Israel	*N* = 44 (M: 27, F: 17; age: 70.5, 63–87*)	*N* = 10 (3 y) *N* = 6 (4 y) *N* = 2 (5 y)	3 y 4 y 5 y	NR	NR	NR	NR	46.0 (16–74)*	3 y: 16.0 (9–57)* 4 y: 14.0 (6–74)* 5 y: 8.0 (6–10)*	41.5 (15–93)*	3 y: 15.5 (8–59)* 4 y: 14.5 (4–28)* 5 y: 11.0 (6–16)*
Present study	Retrospective, monocenter clinical trial	Italy	*N* = 49 (M: 30, F: 19; age: 72.8 ± 6.9)	*N* = 35	3 y	23.5 ± 6.3	12.8 ± 7.9	12.7 ± 4.3	5.8 ± 3.9	58.6 ± 17.1	40.8 ± 18.0	38.0 ± 14.8	18.7 ± 13.3

^#^Refer to the same cohort followed over time (registration no: NCT01827904). *Median, range. ^§^Estimated marginal mean with 95% CI. BL, baseline; CI, confidence interval; CRST, clinical rating scale for tremor; ET, essential tremor; F, females; FU, follow-up; M, males; MRgFUS, magnetic resonance-guided focused ultrasound; N, number; NA, not assessed; NR, not reported; QoL, quality of life; QUEST, Quality of Life in Essential Tremor Questionnaire; y, years.

## Discussion

This retrospective report of 49 patients who underwent unilateral MRgFUS Vim thalamotomy for medically refractory ET, with follow-up data for up to 3 years in 35 of them, documented an overall consistent improvement in the tremor scores on the treated side, the impairment due to tremor, the overall tremor, and the subjective experience of tremor, as well as tremor-related quality of life. Some patients reported reappearance of tremor during follow-up, of whom two were retreated with success. We considered the first case as a ‘technical failure’ because the patient experienced an ‘early’ recurrence of tremor, in the absence of a detectable lesion on MRI. At variance, in the second patient, who exhibited a ‘late’ recurrence of tremor even in the presence of the lesion, it is conceivable that the diminished efficacy was, at least in part, due to worsening of ET because of its natural course.

There are robust data in the literature, supported by meta-analyses, demonstrating the sustained efficacy of MRgFUS treatment at short-term follow-up [i.e., 6 months, 1 year; (5, 6)]. On the other hand, limited and diverse data are accessible for a longer-term follow-up (i.e., > 3 years), as indicated in Table 3. Of the 115 patients with ET, for whom there is a follow-up of at least 3 years, approximately one-third of them come from our cohort. When comparing our data to those previously published, the tremor improvement in our cohort was consistent to that in previously reported ones.

It can be pointed out that this is still a relatively shorter follow-up compared to that of alternative neurosurgical procedures, such as deep brain stimulation, radiofrequency and radiosurgery ablation of the Vim (20, 21). Considering that the MRgFUS literature reports 5-year follow-up data only for 57 patients, there is a need for longer studies to confirm the duration of its effects for longer time periods. In this context, our follow-up data may offer interesting insight, compared to the brief history (i.e., around 10 years) of MRgFUS thalamotomy for refractory ET treatment.

More severe side effects occurred in a minority of treated patients. Mild adverse events were common, but transitory or rapidly improving in most of the cases. The most common ones, in line with previous literature (22) were mild ataxia, dysarthria and paresthesia, which are related to the proximity of the Vim to other thalamic nuclei and the internal capsule. Some patients also reported side effects related to the use of corticosteroids, which are routinely prescribed in our center on the day of the treatment and the following 2–3 weeks to reduce edema secondary to the procedure. These side effects led to a modification of the corticosteroid protocol. Nowadays we administer the same high dose of steroid on the day of the procedure followed by a shortened tapering period (i.e., prednisolone 25 mg for 3 days, then 12.5 mg for 2 days).

The routine use of corticosteroid may account for the significantly lower rate of adverse events, in particular sensory ones, and/or - if present - their rapid resolution (< 15 days) in most cases, when compared to other studies (23). Moreover, the use of steroid may explain the why our lesion volume at 1-month follow-up is much smaller when compared to that reported by other studies that used the same method to measure the size of the lesion (2, 23). We speculate that premedication and an immediate post-procedure protocol with high-dose steroid could mitigate the development of vasogenic edema in the outermost zone [zone III of Wintermark; (24, 25)] thereby explaining the relatively low incidence of adverse effects in the postoperative period. Moreover, it is conceivable that corticosteroids might have reduced cytotoxic edema (zone II of Wintermark) and contributed to reduce the size of the lesion at 1-month follow-up [zone I + zone II of Wintermark; (24, 25)]. Along this line, larger lesion size at 3 months was reported to be heralded by increased edema in the acute phase (26). Admittedly, proving this hypothesis is challenging due to the lack of comparative studies and the limited radiological follow-up data of our cohort, but we consider this is a starting point for future studies. Indeed, quantitative automated methods were not employed and the intrinsic limitations in the methods used to estimate the lesion size might have influenced our findings. Also, we did not systematically assess the lesion size at longer follow-ups. Regardless of the reasons why our lesions appear smaller, the lesion size did not seem to affect our clinical outcomes that are comparable with the literature. This finding is in line with the hypothesis that small lesion size seems not to affect the treatment’s efficacy, as previously reported (14, 27). However, there is no consensus on the use of corticosteroids among centers, and its significance should be better investigated in future multicenter studies.

Finally, SDR was on average high in our patients in that it ranged from 0.41 to 0.75. Some Authors suggest that SDR > 0.45 should predict MRgFUS treatment success and side effects (28), but other studies reported contrasting findings, in that SDR was reported not to influence clinical outcome (2, 29).

### Strength and limitations

The main strength of our study is that it reports a real-world clinical experience that confirms the generalizability of the data on the efficacy of MRgFUs thalamotomy that was previously documented in various reports.

We acknowledge some limitations of this report. First, the study was retrospective and the unblinded evaluation of the patients at different follow-ups carried the risk of positive reporting bias both by observer and patients. Second, the loss of some patients to follow-up because they came from other regions of Italy, and/or the COVID-19 pandemics, might have influenced the statistical analysis, but results did not change when examining patients with 3-year follow-up. Third, the small number of patients impeded the exploration of factors potentially influencing clinical outcomes. Fourth, we did not explore the spread of the lesion to other structures in single patients reporting side effects and did not systematically perform MRI tractography, which was reported to improve MRgFUS targeting, in all the patients (30). Finally, our review of previous studies was not systematic, as it was meant to provide an updated overview of the outcomes of unilateral MRgFUS thalamotomy in medically refractory ET, with long-term follow-ups. Future studies should better explore whether the site and the size of the lesion, as well as the involvement of specific tracts according to tractography predict side effects and their duration.

## Data availability statement

The raw data supporting the conclusions of this article will be made available by the authors, without undue reservation.

## Ethics statement

The studies involving humans were approved by the Ethical Committee of the Veneto Region South-West Area at the Verona University Hospital – CET-ASOV. The studies were conducted in accordance with the local legislation and institutional requirements. The participants provided their written informed consent to participate in this study.

## Author contributions

ST: Writing – review & editing, Writing – original draft, Project administration, Methodology, Investigation, Formal analysis, Data curation, Conceptualization. FP: Writing – review & editing, Writing – original draft, Methodology, Investigation, Formal analysis, Data curation, Conceptualization. TB: Writing – review & editing, Methodology, Investigation, Data curation. GB: Writing – review & editing, Methodology, Investigation, Data curation. ML: Writing – review & editing, Methodology, Investigation, Data curation. RF: Writing – review & editing, Supervision, Project administration, Methodology, Investigation, Funding acquisition, Data curation, Conceptualization. EM: Writing – review & editing, Writing – original draft, Methodology, Investigation, Formal analysis, Data curation. PP: Writing – review & editing, Methodology, Investigation, Data curation. MAT: Writing – review & editing, Methodology, Investigation, Data curation. EZ: Writing – review & editing, Methodology, Investigation, Data curation. CZ: Writing – review & editing, Methodology, Investigation, Data curation. CC: Writing – review & editing, Supervision, Methodology, Data curation. AN: Writing – review & editing, Supervision, Methodology, Data curation. BP: Writing – review & editing, Supervision, Methodology, Data curation. FS: Writing – review & editing, Supervision, Methodology. BB: Writing – review & editing, Supervision, Project administration, Methodology, Conceptualization. MHT: Writing – review & editing, Supervision, Project administration, Methodology, Conceptualization. SM: Writing – review & editing, Supervision, Project administration, Methodology, Conceptualization. GR: Writing – review & editing, Writing – original draft, Supervision, Software, Project administration, Methodology, Investigation, Data curation, Conceptualization.
